# Prognostic Factors of Morbimortality in Patients Treated for Xanthogranulomatous Pyelonephritis

**DOI:** 10.31486/toj.23.0002

**Published:** 2023

**Authors:** Luis R. García-Chairez, Jose Ivan Robles-Torres, Roberto Alejandro Ríos-Palacios, Joana Valeria Enrriquez-Avila, Hector Erasmo Alcocer-Mey, Daniel Eduardo Cervantes-Miranda, Adrián Gutierrez-González

**Affiliations:** Department of Urology, Hospital Universitario Dr. José Eleuterio González, Universidad Autónoma de Nuevo León, Monterrey, Nuevo León, México

**Keywords:** *Nephrectomy*, *prognosis*, *pyelonephritis–xanthogranulomatous*

## Abstract

**Background:** Nephrectomy is the treatment for xanthogranulomatous pyelonephritis (XGP), but the surgery is often technically complex and associated with a high incidence of postoperative complications. The objective of this study was to determine factors that can predict the probability of major postoperative complications, admission to intensive care, or mortality.

**Methods:** We conducted a retrospective observational study of patients with XGP who underwent simple nephrectomy in a tertiary care hospital in Mexico from 2015 to 2022. We analyzed preoperative and transoperative variables to determine their relationship with postoperative complications.

**Results:** A total of 39 patients with a mean age of 44.33 ± 12.6 years were included. In the comparative analysis of the variables, we found a significant difference in the amount of intraoperative bleeding between the types of surgical approaches—a median of 1,200 mL with the transperitoneal approach vs 525 mL with the retroperitoneal approach (*P*=0.02)—but we found no significant differences in the need for blood transfusion or other complications associated with surgical approach. In both the univariate and multivariate analyses, patients with positive urine cultures prior to surgery had a higher rate of complications requiring surgical, endoscopic, or radiologic intervention. No significant differences in outcomes were found between patients who underwent early vs delayed nephrectomy.

**Conclusion:** The surgical approach for nephrectomy, transperitoneal or retroperitoneal, and early vs delayed surgery did not affect postoperative complications in our patients with XGP. However, the presence of positive urine cultures prior to surgery was associated with major complications.

## INTRODUCTION

Xanthogranulomatous pyelonephritis (XGP), a rare and aggressive condition characterized by chronic infection of the kidney and surrounding tissues with extensive fibrosis and renal parenchyma destruction, can potentially result in a nonfunctional kidney.^1^ XGP has a very low incidence, occurring in 0.6% to 1.4% of chronic pyelonephritis cases.^2^

XGP is commonly called the great imitator because of the vague and nonspecific clinical manifestations and radiologic findings.^3^ The most common symptoms are malaise and abdominal pain, predominantly in the renal fossa. Confirmatory diagnosis is histopathologic. Macroscopically, the kidney is yellowish, enlarged, and generally associated with lithiasis. Microscopic findings show macrophages with abundant lipids, typically called foamy histiocytes, that explain the characteristic color, accompanied by necrosis and the infiltration of plasma cells and leukocytes.^4^

Imaging studies—contrast-enhanced computed tomography is the method of choice—provide evidence for a presumptive diagnosis of XGP, showing the extension of disease and the involvement of surrounding organs.^5^ Typical findings include a radiologic characteristic called the bear paw sign^6^ that represents the loss or replacement of renal parenchyma by necrotic areas and xanthomas, in addition to the dilation of the renal calyces or hydronephrosis accompanied by peripheral contrast enhancement. Malek and Elder developed an XGP classification based on disease extension: (1) stage I, limited to the renal capsule; (2) stage II, confined within the Gerota fascia; and (3) stage III, involvement of tissue outside the Gerota fascia.^7^

The treatment of choice is nephrectomy, but the procedure can be technically challenging because of the extensive fibrosis and involvement of adjacent structures, including organs and blood vessels. The surgical team needs to be prepared to remove nearby organs and have vascular grafts available in case they are necessary. Although rare, the main complication is sepsis after the surgical procedure. Leoni et al found that surgical complications occurred in 50% of cases, with sepsis, wall abscess, psoas abscess, and hemorrhage being the most common.^8^ A 2023 systematic review reported a weighted pooled mortality rate of 1,436 deaths per 100,000 patients with XGP.^9^

The literature about XGP is limited. Currently, no standardized management is described for this population. Surgical complications are relatively common, but to our knowledge, no predictors of complications have been described. The identification of patients with a high risk of major complications is crucial to establish therapeutic modalities accordingly. The objective of this study was to determine the factors associated with major complications in patients undergoing nephrectomy because of XGP.

## METHODS

We conducted a retrospective observational study of patients diagnosed with XGP who underwent simple nephrectomy at a tertiary care hospital in Mexico from 2015 to 2022. All patients had a confirmed histopathologic diagnosis of XGP from surgical specimen. The protocol was approved by the Ethics and Research Committee of the Universidad Autónoma de Nuevo León (UR21-00003).

We reviewed clinical records to obtain sociodemographic data of age, sex, and body mass index; comorbidities; and biochemical variables such as hemoglobin, white blood cell count, and preoperative creatinine. Radiologic features, including the presence of pyonephrosis, kidney abscess, stone size and location, and Malek and Elder classification^7^ were assessed. We also evaluated intraoperative variables: type of surgical approach (transperitoneal or retroperitoneal), total bleeding, surgical time, need for transfusion, length of hospital stay, and perioperative complications up to 10 days after the surgical procedure.

For this study, early nephrectomy was defined as occurring ≤3 days postadmission, and delayed nephrectomy was defined as occurring ≥4 days postadmission. Complications were classified as minor and major according to the Clavien-Dindo classification.^10^ Major complications were defined as Clavien-Dindo ≥grade III: complications that required surgical, endoscopic, or radiologic intervention (grade III); life-threatening complications that required admission to the intensive care unit (ICU) (grade IV); and mortality (grade V).

Categorical variables are presented as frequency and percentage. Numeric variables are presented as mean and standard deviation or median and interquartile range, and Kolmogorov-Smirnov test was used to classify numeric variables according to the distribution of data. For categorical variables, chi-square test and Fisher exact test were used to determine factors associated with major complications. The Mann-Whitney *U* test was used for numeric variables with nonnormal distribution.

We performed a subanalysis to determine the prognostic factors associated with the presence of bacterial growth in urine. Variables with statistical significance in the univariate analysis were included in a multivariate logistic regression. Statistical significance was established at *P*<0.05.

## RESULTS

A total of 39 patients were included in the analysis (Table 1). The mean age of the population was 44.33 ± 12.6 years, and XGP was more frequent in females (n=27, 69.2%). The mean body mass index of the enrolled patients was 26.43 ± 4.41, classified as overweight. Among the comorbidities, the most frequent were diabetes mellitus and recurrent urinary tract infections, both with a prevalence of 38.5% of the study subjects, followed by hypertension in 35.9%. At admission to the hospital, the most common clinical presentation was symptomatic urinary tract infection in 18 patients (46.1%); only 3 patients (7.7%) presented with urosepsis. Urolithiasis was present in 27 patients (69.2%). Stone in the uretero-pelvic junction was the most common in our population (n=22, 56.4%), followed by staghorn stone in 17 patients (43.6%).

**Table 1.** Characteristics of the Study Population, n=39

Variable	Value
Demographics	
Age, years, mean ± SD	44.33 ± 12.6
Female	27 (69.2)
Right kidney laterality	15 (38.4)
Body mass index, kg/m^2^, mean ± SD	26.43 ± 4.41
Clinical presentation	
Symptomatic urinary tract infection	18 (46.1)
Urosepsis	3 (7.7)
Comorbidities	
Diabetes mellitus	15 (38.5)
Recurrent urinary tract infections	15 (38.5)
Hypertension	14 (35.9)
Chronic kidney disease	9 (23.1)
Radiologic characteristics	
Kidney abscess	19 (48.7)
Pyonephrosis	7 (17.9)
Staghorn calculi	17 (43.6)
Multiple stones	10 (25.6)
Stone in uretero-pelvic junction	22 (56.4)
Laboratory findings	
Anemia (hemoglobin <12 g/dL)	29 (74.3)
Leukocytosis (WBC >12,000/μL)	19 (48.7)
Thrombocytopenia (platelets <150,000/L)	4 (10.2)
Elevated creatinine (creatinine >1.2 mg/dL)	11 (28.2)
Hyperglycemia (glucose >200 mg/dL)	20 (51.3)
Glomerular filtration rate, mL/min, median [IQR]	88 [53.7-109.5]
Malek and Elder^7^ classification	
Stage I, Limited to renal parenchyma	12 (30.8)
Stage II, Extension to perirenal space	11 (28.2)
Stage III, Extension to pararenal space	16 (41.0)
Microbiology findings	
Positive urine culture	19 (48.7)
Positive kidney culture	23 (59.0)
ESBL-producing agent in urine	5 (12.8)
ESBL producing agent in kidney	6 (15.4)
Nephrectomy approach	
Transperitoneal	23 (59.0)
Retroperitoneal	16 (41.0)
Perioperative findings	
Hospital length of stay, days, median [IQR]	9 [1.0-14.5]
Surgical time, minutes, median [IQR]	197 [147.5-230.0]
Total reported bleeding, mL, median [IQR]	700 [325-1,400]
Need for transfusion	31 (79.5)
Complications (Clavien-Dindo classification^10^)^a^	
Grade I	4 (10.2)
Grade II	12 (30.8)
Grade IIIa	1 (2.6)
Grade IIIb	11 (28.2)
Grade IVa	5 (12.8)
Grade IVb	0 (0.0)
Grade V	3 (7.7)
Clinical outcome	
Intensive care unit admission	7 (17.9)
Mortality	3 (7.7)

^a^Twenty patients had Clavien-Dindo ≥grade III complications. Of the 7 patients who required intensive care unit admission, 5 patients are classified here as Clavien-Dindo grade IV, and 2 patients are classified as Clavien-Dindo grade V because they died.

Note: Data are presented as n (%), unless otherwise indicated.

ESBL, extended-spectrum beta-lactamase; IQR, interquartile range; WBC, white blood cells.

Twelve patients (30.8%) had disease limited to the kidney, 11 patients (28.2%) had perinephric extension, and 16 patients (41.0%) had paranephric extension.

Major complications (Clavien-Dindo ≥grade III^10^) were reported in 20 patients (51.3%). Injuries to the intestine were the most common, with the colon the most commonly affected site (20.5%), followed by intra-abdominal abscess requiring percutaneous drainage (12.8%) (Table 2). Ten of these patients also had 2 or more Clavien-Dindo grade IV or V complications: 7 required ICU admission, and 3 died during the early postoperative period.

**Table 2.** Major Complications (Clavien-Dindo Grade ≥III^10^), n=39

Complication	n (%)
Grade III: Required surgical, endoscopic, or radiologic intervention	20 (51.3)
Colon injury	8 (20.5)
Intra-abdominal abscess	5 (12.8)
Duodenal injury	2 (5.1)
Vascular injury	2 (5.1)
Wound infection	2 (5.1)
Spleen injury	1 (2.6)
Grade IV: Required intensive care unit admission	7 (17.9)
Grade V: Mortality	3 (7.7)

Note: Ten patients had 2 or more major complications.

Fifty-nine percent of patients underwent nephrectomy through a transperitoneal approach, compared to 41.0% who had a retroperitoneal approach (Table 3). In the evaluation of outcomes between the 2 approaches, no significant differences were found in hospital length of stay (*P*=0.088), surgical time (*P*=0.063), need for transfusion (*P*=0.563), major complications (*P*=0.151), ICU admission (*P*=0.913), or mortality (*P*=0.557). However, a significant difference was observed in total reported bleeding: a median of 1,200 mL in the transperitoneal group vs 525 mL in the retroperitoneal group (*P*=0.02).

**Table 3.** Outcomes Stratified by Surgical Approach

Outcome	Transperitoneal Approach, n=23	Retroperitoneal Approach, n=16	*P* Value	Odds Ratio (95% CI)
Perioperative findings				
Hospital length of stay, days, median [IQR]	13 [7.5-15]	5 [3.5-11.5]	0.088^a^	NA
Surgical time, minutes, median [IQR]	215 [183.0-240.0]	152.5 [3.5-11.5]	0.063^a^	NA
Total reported bleeding, mL, median [IQR]	1,200 (500-1,850)	525 (250-700)	0.02^a^	NA
Need for transfusion	19 (82.6)	12 (75.0)	0.563^b^	0.632 (0.132-3.015)
Complications (Clavien-Dindo classification^10^)				
Grade III: Required surgical, endoscopic, or radiologic intervention	14 (60.9)	6 (37.5)	0.151^b^	0.386 (0.104-1.435)
Grade IV: Required intensive care unit admission	4 (17.4)	3 (18.8)	0.913^c^	1.096 (0.209-5.735)
Grade V: Mortality	1 (4.3)	2 (12.5)	0.557^c^	3.143 (0.260-37.991)

^a^Mann-Whitney *U* test.

^b^Chi-square test.

^c^Fisher exact test.

Notes: Data are presented as n (%), unless otherwise indicated. Ten patients had 2 or more major complications.

IQR, interquartile range; NA, not applicable.

In the comparison of outcomes between patients who underwent early vs delayed nephrectomy (Table 4), no significant differences were found in hospital length of stay (*P*=0.914), surgical time (*P*=0.633), total reported bleeding (*P*=0.21), need for transfusion (*P*=0.821), major complications (*P*=0.798), ICU admission (*P*=0.342), or mortality (*P*=0.187).

**Table 4.** Outcomes Stratified by Surgical Timing

Outcome	Early Nephrectomy, n=11	Delayed Nephrectomy, n=28	*P* Value	Odds Ratio (95% CI)
Perioperative findings				
Hospital length of stay, days, median [IQR]	7 [4.5-14.0]	10 [4.0-14.5]	0.914^a^	NA
Surgical time, minutes, median [IQR]	215 [167.5-235.0]	193.5 [140.0-225.0]	0.633^a^	NA
Total reported bleeding, mL, median [IQR]	1,000 [475-2,400]	650 [275-1,200]	0.21^a^	NA
Need for transfusion	9 (81.8)	22 (78.6)	0.821^b^	1.227 (0.207-7.265)
Complications (Clavien-Dindo classification^10^)				
Grade III: Required surgical, endoscopic, or radiologic intervention	6 (54.5)	14 (50.0)	0.798^b^	1.2 (0.296-4.862)
Grade IV: Required intensive care unit admission	3 (27.3)	4 (14.3)	0.342^c^	2.25 (0.412-12.284)
Grade V: Mortality	2 (18.2)	1 (3.6)	0.187^c^	6.0 (0.485-74.289)

^a^Mann-Whitney *U* test.

^b^Chi-square test.

^c^Fisher exact test.

Notes: Data are presented as n (%), unless otherwise indicated. Ten patients had 2 or more major complications. Early nephrectomy occurred ≤3 days postadmission; delayed nephrectomy occurred ≥4 days postadmission.

IQR, interquartile range; NA, not applicable.

In the univariate analysis evaluating outcomes between patients with positive and negative urine cultures, hospital length of stay (*P*=0.019) was longer for patients with positive urine cultures, and major complications (*P*=0.006, odds ratio [OR] 6.181, 95% CI 1.561-27.71) and admission to the ICU (*P*=0.031, OR 8.769, 95% CI 1.942-81.671) occurred more frequently in patients with positive cultures. In the multivariate analysis, only major complications showed a statistically significant difference for patients with positive urine cultures (*P*=0.02, OR 2.1, 95% CI 1.232-3.143) (Table 5).

**Table 5.** Prognostic Factors Associated With the Presence of Bacterial Growth in Urine

			Univariate Analysis		Multivariate Analysis	
Variable	Negative Culture, n=20	Positive Culture, n=19	*P* Value	Odds Ratio (95% CI)	*P* Value	Odds Ratio (95% CI)
Comorbidities						
Diabetes mellitus	7 (35.0)	8 (42.1)	0.648^a^	1.351 (0.370-4.925)		
Hypertension	6 (30.0)	8 (42.1)	0.431^a^	1.697 (0.453-6.356)		
Chronic kidney disease	1 (5.0)	8 (42.1)	0.008^b^	13.818 (1.520-125.648)	0.057	10.273 (0.931-113.373)
Recurrent urinary tract infections	6 (30.0)	9 (47.4)	0.265^a^	2.1 (0.565-7.811)		
Laboratory findings						
Anemia (hemoglobin <12 g/dL)	13 (65.0)	16 (84.2)	0.17^a^	2.872 (0.617-13.366)		
Leukocytosis (WBC >12,000/μL)	8 (40.0)	11 (57.9)	0.264^a^	2.063 (0.575-7.393)		
Thrombocytopenia (platelets <150,000/L)	1 (5.0)	3 (15.8)	0.267^b^	3.563 (0.337-37.687)		
Elevated creatinine (creatinine >1.2 mg/dL)	3 (15.0)	8 (42.1)	0.08^b^	4.121 (0.894-19.001)		
Hyperglycemia (glucose >200 mg/dL)	10 (50.0)	10 (52.6)	0.869^a^	1.111 (0.316-3.904)		
Perioperative findings						
Hospital length of stay, days, median [IQR]	5 [3.0-11.5]	13 [8.0-15.0]	0.019^c^	NA	0.208	NA
Surgical time, minutes, median [IQR]	172.5 [131.5-229.0]	210 [180.5-230.0]	0.141^c^	NA		
Total reported bleeding, mL, median [IQR]	700 [275-1,400]	800 [400-1,450]	0.411^c^	NA		
Need for transfusion	15 (75.0)	16 (84.2)	0.476^a^	1.778 (0.361-8.764)		
Complications (Clavien-Dindo classification^10^)						
Grade III: Required surgical, endoscopic, or radiologic intervention	6 (30.0)	14 (73.7)	0.006^a^	6.181 (1.561-27.71)	0.02	2.1 (1.232-3.143)
Grade IV: Required intensive care unit admission	1 (5.0)	6 (31.6)	0.031^b^	8.769 (1.942-81.671)	0.459	2.668 (0.199-35.820)
Grade V: Mortality	0 (0)	3 (15.8)	0.106^b^	0.444 (0.308-0.640)		

^a^Chi-square test.

^b^Fisher exact test.

^c^Mann-Whitney *U* test.

Notes: Data are presented as n (%), unless otherwise indicated. Ten patients had 2 or more major complications.

IQR, interquartile range; WBC, white blood cells.

## DISCUSSION

XGP is an uncommon chronic kidney infection, and the treatment of choice—nephrectomy—is associated with many challenges.^11^

According to previous studies, the most common age at presentation for XGP is between the fourth and sixth decades of life, and the condition shows a predilection for females.^12^ Our study shows similar female and age predilections, with a population composed of 69.2% females and a mean age of 44.3 years. Loffroy et al reported urolithiasis in 72.7% of their patients and intraparenchymatous collection in 45.5% of patients.^5^ Similarly, urolithiasis was present in 69.2% of our population, and 48.7% of our patients presented with renal abscess.

Studies have reported a high rate of postoperative complications. Leoni et al reported major postoperative surgical complications in 50% of patients and a mortality rate of 10%,^8^ similar to our major complication rate of 51.3% and our mortality rate of 7.7%.

In a 2013 study, León Mar et al evaluated the prognostic factors of morbidity and mortality in 32 patients treated for XGP.^13^ They described complications associated with nephrectomy according to the Clavien-Dindo classification^10^ and reported complications in 28.1% of patients. León Mar et al concluded that the presence of leukocytes >12,000/μL and extension of the disease according to the Malek and Elder classification^7^ ≥stage II were predictive factors of complications in patients treated with nephrectomy.^13^ In our study, a significant difference was observed in bleeding (*P*=0.02) in the comparison of the transperitoneal vs retroperitoneal approach. Also, patients with positive urine cultures were associated with more major complications in the multivariate analysis (*P*=0.02).

As previously mentioned, because nephrectomy is a challenging and technically complex procedure, identification of perioperative factors is necessary to prevent and manage potential complications accordingly. Montelongo-Rodríguez et al reported that anemia and the presence of renal abscess were associated with major complications.^14^ Although anemia and renal abscess were present in our study population, we did not purposefully look for an association between these factors and the occurrence of major complications.

Unlike previously published studies, we found that the presence of positive urine cultures prior to surgery was a prognostic factor for major complications. Although not all patients with XGP have positive urine cultures, our prevalence differs from the prevalence of positive cultures reported by Petca et al.^15^ In their study, almost two-thirds (62.06%) of patients had a positive urine culture, while our prevalence was 48.7%, with 12.8% of these patients having an extended-spectrum beta-lactamase–producing agent in the urine.

Our study has several limitations. The retrospective design and the sample size are factors that may contribute to bias in our results. Additionally, the absence of therapeutic algorithms for XGP means that medical management in this population might be arbitrary and based on individual physician criteria, including surgical approach and the appropriate time for surgery. Another important limitation of this study is that we did not evaluate the factors associated with complications.

## CONCLUSION

Surgical approach (transperitoneal or retroperitoneal) and surgical timing (early or delayed) for nephrectomy in patients with XGP had no effect on postoperative complications in our study population. However, the presence of positive urine cultures prior to surgery was associated with major complications in our population. Further prospective and more extensive studies are needed to identify the factors associated with major complications in patients with XGP.

## Notes

*This article meets the Accreditation Council for Graduate Medical Education and the American Board of Medical Specialties Maintenance of Certification competencies for Patient Care, Medical Knowledge, and Practice-Based Learning and Improvement.*
